# Genetic Determinants in MRSA Carriage and Their Association with Decolonization Outcome

**DOI:** 10.1007/s00284-023-03581-w

**Published:** 2024-01-13

**Authors:** Annette C. Westgeest, Emile F. Schippers, Sigrid Rosema, Monika A. Fliss, Ed J. Kuijper, Romy D. Zwittink, Mariëtte Lokate, Marjan Wouthuyzen-Bakker, Merel M. C. Lambregts, Erik Bathoorn

**Affiliations:** 1 Department of Infectious Diseases, Leiden University Medical Center, Albinusdreef 2, 2333 ZA Leiden, The Netherlands; 2 Department of Internal Medicine, Haga Teaching Hospital, Els Borst-Eilersplein 275, 2545 AA The Hague, The Netherlands; 3 Department of Medical Microbiology and Infection Prevention, University of Groningen, University Medical Center Groningen, Hanzeplein 1, 9713 GZ Groningen, The Netherlands; 4 Center for Infectious Disease Control, National Institute for Public Health and the Environment (RIVM), Antonie Van Leeuwenhoeklaan 9, 3721 MA Bilthoven, The Netherlands

## Abstract

Methicillin-resistant *Staphylococcus aureus* (MRSA) colonization increases the risk of infection. Response to decolonization treatment is highly variable and determinants for successful decolonization or failure of eradication treatment are largely unknown. Insight into genetic predictors of eradication failure is potentially useful in clinical practice. The aim of this study was to explore genetic characteristics that are associated with MRSA decolonization failure. This cohort study was performed in a tertiary care hospital in the Netherlands. Patients with ≥ 1 positive MRSA culture from any site and with available whole -genome sequencing data of the MRSA isolate between 2017 and 2022 were included. Lineages, resistance, and virulence factors were stratified by MRSA decolonization outcome. In total, 56 patients were included: 12/56 (21%) with treatment failure and 44/56 (79%) with successful decolonization (with or without preceding treatment). A significant association was found between ciprofloxacin-resistant lineages and failure of eradication (OR 4.20, 95%CI 1.11–15.96, *P* = 0.04). Furthermore, livestock-associated MRSA and the major community-associated MRSA lineages ST6-t304 and ST8-t008 were associated with successful eradication treatment or spontaneous clearance. In conclusion, this explorative study showed a higher eradication failure rate in complicated MRSA carriers with ciprofloxacin-resistant MRSA lineages, which are predominantly healthcare-associated. Further studies are warranted to confirm the higher eradication failure risk of ciprofloxacin-resistant lineages, and identify the underlying mechanisms.

**Supplementary Information:**

The online version contains supplementary material available at 10.1007/s00284-023-03581-w.

## Introduction

Methicillin-resistant *Staphylococcus aureus* (MRSA) is a global health threat with high morbidity and mortality rates [1]. Colonization with MRSA leads to increased infection rates of up to 25% [2, 3]. The Netherlands has one of the lowest levels of endemic MRSA in the world [4]. This low prevalence is for a large part attributed to a successful ‘search and destroy’ policy aiming at MRSA carriage, that has been executed for over three decades [5]. This policy consists of screening and pre-emptive strict isolation of patients with increased risk of MRSA carriage when hospitalized and subsequent decolonization treatment when carriage is found. Response to decolonization treatment is highly variable; in some patients, eradication treatment fails despite multiple attempts, in others colonization is self-limiting without treatment [6, 7]. Spontaneous clearance or persistent carriership is driven by a complex host–pathogen interaction, which is largely unraveled. Furthermore, antimicrobial treatment (i.e., eradication therapy) adds to this complex interaction, and introduces pharmacodynamic and pharmacokinetic effects. In summary, patient characteristics, antibiotic regimen, and isolate characteristics are all considered to contribute to decolonization treatment outcomes [7–9].

Different MRSA clones have emerged throughout the world with a high variety in virulence factors [10]. The rapid developments in the field of genetic diagnostics, especially whole-genome sequencing (WGS), have expanded the knowledge of the complexity and heterogeneity of this pathogen. MRSA strains produce a broad range of virulence factors, such as toxins, immune evasion factors, and adhesion proteins [11]. These virulence determinants are mostly carried on mobile genetic elements (MGEs), such as pathogenicity islands, plasmids, or bacteriophages [3]. Furthermore, virulence determinants can vary between hospital-associated, community-associated, and livestock-associated (LA) MRSA strains [12].

WGS of MRSA strains has been deployed extensively for infection control purposes. It has proven to be of great value in the epidemiology and outbreak management of MRSA [13]. In addition, WGS allows for molecular characterization of isolates by identifying clinically relevant genetic determinants that can help to predict response to decolonization treatment. So far, microbial genomics is not yet broadly applied to identify determinants related to MRSA eradication treatment outcome [14]. As an example, the presence of Panton-Valentine leucocidin (PVL) genes and genes associated with mupirocin resistance were associated with successful eradication outcome [9, 15]. A recent study elaborated on genetic factors and carriage duration, and showed a potential role of bacteriophage-related chemotaxis inhibitory protein encoded by *chp* [8]. Insight into genetic predictors of eradication failure is potentially useful in clinical practice. Ultimately, differentiating between MRSA carriers that will benefit from an eradication treatment and carriers more prone to eradication failure may enable personalized medicine.

In this explorative pilot cohort study, we evaluated genomic characteristics that are associated with MRSA decolonization failure. This was established by linking WGS data of MRSA isolates to clinical patient characteristics.

## Methods

This cohort study was conducted at the University Medical Center Groningen, a tertiary hospital in the Northern part of the Netherlands, between 2017 and 2022. The prevalence of MRSA carriage in the Netherlands during this time was < 1%. During these years, genetic analyses of first MRSA isolates (both from carriage and infection) had been performed in all index patients and most of the healthcare workers, for the purpose of surveillance and outbreak management. Genetic analysis was not performed in healthcare workers who were positive at their pre-employment screening nor in positive family contacts of index patients. All patients (both adults and children) and healthcare workers of whom WGS of an MRSA isolate was performed were retrospectively identified and were screened to meet the selection criteria. Healthcare workers will be also addressed as ‘patients’ from now on in this manuscript, since they were treated as patients for this matter. Inclusion criteria were ≥ 1 visit to the outpatient infectious diseases clinic because of MRSA carriage or infection, ≥ 1 positive MRSA culture from any site, and available WGS data of the MRSA isolate. Exclusion criterion was the absence of follow-up cultures. Only the first available MRSA isolate per patient was included in the analysis. The patients had been assessed by the outpatient clinicians using protocols based on the national MRSA eradication guideline [16]. This includes in case of an MRSA infection, adequately treating the infection first, and subsequently screen for persistent colonization.

### Data Collection

Clinical data were extracted from the electronic patient files. This included demographics, complicated versus uncomplicated carriage, treatment regimen, duration of therapy, and follow-up cultures. MRSA culture results were extracted from the laboratory information system. This included initial and follow-up MRSA cultures, including minimal inhibitory concentrations (MICs) of antibiotics, phenotypic susceptibility results, and WGS results.

### Microbiological Methods

Culturing using BHI broth with 2.5% saline and MRSAid chromagar (bioMérieux, Lyon, France), susceptibility determination by automated susceptibility testing by VITEK2 (bioMérieux, Lyon, France), and cefoxitin disk diffusion were performed according to the Dutch Society of Medical Microbiology guideline for laboratory detection of highly resistant microorganisms as part of routine diagnostic procedures [17]. MIC breakpoints and zone diameter breakpoints for resistance and intermediate sensitivity were based on EUCAST criteria [18]. The isolates were identified as *S. aureus* by matrix-assisted laser desorption/ionization–time of flight mass spectrometry (Bruker Daltonics, Billerica, US). First MRSA isolates per patient were genotypically confirmed by Xpert MRSA NxG based on the detection of the *mecA* or *mecC* targets (Cepheid, Sunnyvale, US).

A total DNA extraction for whole-genome sequencing was performed directly from colonies of the respective isolates using the Ultraclean Microbial DNA Isolation Kit (MO BIO Laboratories, Carlsbad, CA, US) according to the manufacturer’s protocol. DNA concentrations were determined using a Qubit® 2.0 fluorometer and the dsDNA HS and/or BR assay kit (Life Technologies, Carlsbad, CA, US). Subsequently, DNA libraries were prepared using the Nextera XT v2 kit (Illumina, San Diego, CA, US) according to the manufacturer’s instructions. Short-read sequencing was performed with an Illumina MiSeq System generating paired-end reads of 250 bp. De novo assembly of paired-end reads was performed using CLC Genomics Workbench v12.0.1-v20.0.4 (QIAGEN, Hilden, Germany) after quality trimming (Qs ≥ 20) establishing a word size of 29.

Based on next generation sequencing data (ENA project number PRJEB59407), molecular typing was performed using Ridom Seqsphere + v8.3.1 (Ridom, Münster, Germany). Herewith multilocus sequence typing (MLST) ST type was derived and core genome multilocus sequence typing (cgMLST) was performed using a scheme including 1861 alleles [19]. Isolates with a maximum of 24 allelic differences were denominated the same complex type. Antibiotic resistance genes were identified by Resfinder v4.1 (Center for Genomic Epidemiology, Lingby, Denmark). A predefined set of virulence factors were identified using AlereMicroarray schemes in Ridom Seqsphere + v8.3.1 (Ridom, Münster, Germany) [20].

### Definitions

Uncomplicated MRSA carriage was defined as having all of the following features: (i) the presence of MRSA exclusively located in the nose, (ii) no active infection with MRSA, (iii) in vitro susceptibility for mupirocin, (iv) the absence of active skin lesions, (v) the absence of foreign material that connects an internal body site with the outside (e.g., urine catheter, external fixation material), and (vi) no previously failure of decolonization treatment. All other carriage cases were considered complicated colonization. Uncomplicated carriage is advised to be treated with topical therapy (mupirocin topically applied to the nares, disinfecting shampoo) and hygienic measures. In cases of complicated MRSA carriage, additional systemic antimicrobial therapy with a combination of two antibiotic agents is recommended, according to the national guideline [16]. MRSA infection was defined as a positive culture send to the microbiology laboratory from an infected body site as indicated by the treating physician.

Successful decolonization was defined as three consecutive negative MRSA cultures from swabs taken from nose, throat, and perineum, with the cultures obtained at 1-week intervals, without antibiotic usage [16]. For analyses, patients were divided in two groups: patients with failure of eradication treatment (failure group) and patients with successful decolonization with or without preceding treatment (successful decolonization group).

Livestock-associated MRSA was defined based on the Spa-type. The Spa-types *t011, t034, t108, t567, t571, t588, t753, t779, t898, t899, t943, t1184, t1197, t1254, t1255, t1451, t1456, t1457, t2123, t2287, t2329, t2330, t2383, t2582, t2748, t2971, t2974, t3013, t3014, t3053, t3146*, and *t3208* were considered to be associated with livestock [12]. All other Spa-types were considered to be not associated with livestock.

### Statistical Analysis

Data are presented as percentages or proportions for categorical variables and as medians plus interquartile range (IQR) for continuous variables. Univariate analysis was performed using Fisher’s exact test. As this study has an explorative character, no adjustment for multiple testing was done.

## Results

**Fig. 1:**
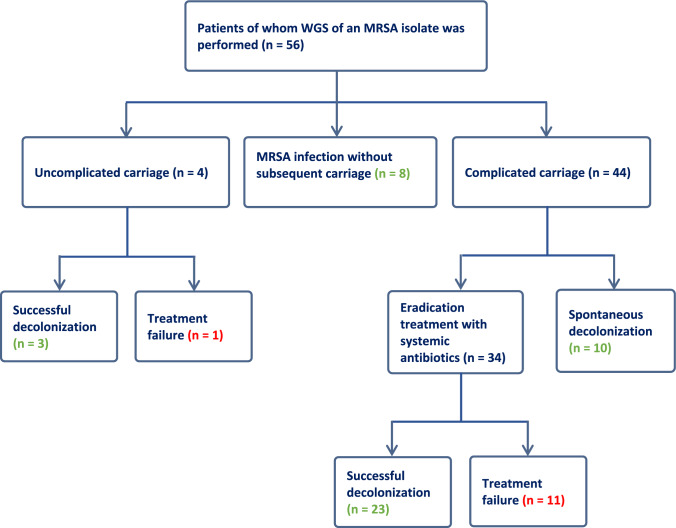
Flow chart. Flow chart of inclusions. MRSA carriage was defined as complicated in 44/56 (79%) patients, of whom 34/44 (77%) received systemic antibiotics as eradication treatment. The other 10/44 (23%) patients with complicated carriage were spontaneously cleared of MRSA before start of the planned eradication treatment. In addition, 8/56 (14%) patients with MRSA infections did not have subsequent MRSA carriage, and 4/56 (7%) patients had uncomplicated carriage. The green numbers represent the successful decolonization group (with or without preceding treatment). The red numbers represent the treatment failure group

During the study period, 181 patients visited the MRSA outpatient clinic. WGS was performed in 56/181 (31%) patients and these were included in the study (Fig. 1). As shown in Fig. 1, there were 12 patients with treatment failure (i.e., one in the uncomplicated carriage group and eleven in the complicated carriage group). All other patients (44) were MRSA negative at the end of follow-up and were defined as successfully decolonized (three in the uncomplicated carriage group, eight with MRSA infection without subsequent carriage, ten with spontaneous decolonization and 23 with successful treatment of complicated carriage). Patient and treatment characteristics of these two groups are depicted in Table 1. In the failure group, one patient out of twelve (8%) had uncomplicated carriage and 11/12 (92%) patients had complicated carriage. The successful decolonization group existed of 33/44 (75%) patients with complicated carriage, 3/44 (7%) patients with uncomplicated carriage, and 8/44 (18%) patients with MRSA infection, without subsequent carriage. Twenty-six out of 44 (59%) patients successfully underwent eradication treatment, in 10/44 (23%) patients colonization resolved spontaneously and 8/44 (18%) were treated for an MRSA infection, without subsequent eradication treatment. Of all 34 patients who underwent eradication treatment for complicated MRSA carriage, 11/34 (32%) had treatment failure. No significant differences in treatment characteristics were found between patients with treatment success and treatment failure (Table 1).

**Table 1:** Baseline and treatment characteristics

Characteristic	Treatment failure	Successful decolonization	*P*
	*n* = 12	*n* = 44	
Sex, male (*n* (%))	6 (50.0)	25 (56.8)	0.75
Age (median (IQR))	23.5 (23)	35.5 (41)	0.11
Complicated carriage	11 (91.7)	33 (75.0)	1.00
Uncomplicated carriage	1 (8.3)	3 (6.8)	1.00
MRSA infection, no subsequent carriage	0	8 (18.2)	n.a
MRSA infection (*n* (%))	3 (25.0)	18 (41.9)	0.34
Treatment regimen (*n* (%))*			0.24
Rifampicin + doxycycline	3/11 (27.3)	9/23 (39.1)	
Rifampicin + cotrimoxazole	4/11 (36.4)	7/23 (30.4)	
Rifampicin + trimethoprim	4/11 (36.4)	3/23 (13.0)	
Rifampicin + clindamycin	0	3/23 (13.0)	
Vancomycin + clindamycin	0	1/23 (4.3)	
Treatment duration (*n* (%))*			
7-day treatment	10/11 (90.9)	19/23 (82.6)	1.00
14-day treatment	1/11 (9.1)	4/23 (17.4)	1.00

*This percentage represents the percentage of the patients who were treated with systemic antibiotics. Patients with uncomplicated carriage, MRSA infection without subsequent carriage, or spontaneous decolonization were not treated with systemic antibiotics

*n.a.* not applicable

### Lineages

**Fig. 2:**
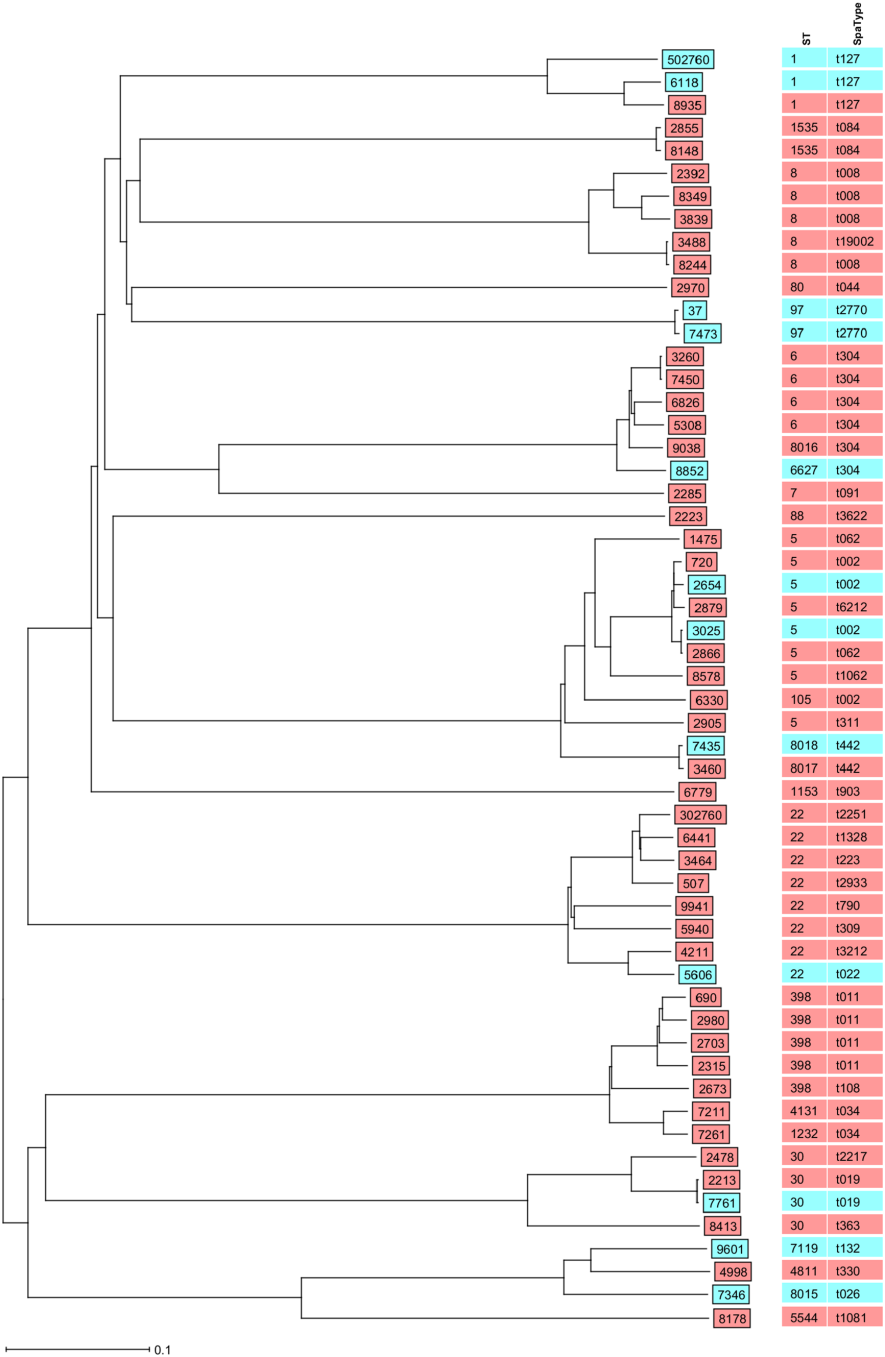
Phylogenetic tree of MRSA isolates. Neighbor-joining tree from SeqSphere software based on curated schema where comparison of 1861 core genes of S. aureus was used. The study isolates from patients who failed on eradication treatment are presented in blue, and from patients with successful decolonization in pink. The corresponding isolate antibiotic susceptibility profiles are shown in supplementary table S1

Among the 56 MRSA isolates, 24 different MLST types were represented. The most predominant MLST types were ST5 (8/56) and ST22 (8/56), followed by ST8 (5/56) and ST398 (5/56) (Fig. 2 and Table S1). The complex types were mostly unique, only seven complex types were represented twice (2615, 4940, 6749, 9359, 10,282, 17,413, 24,737). All isolates (*n* = 7) with livestock-associated Spa-types belonged to clonal complex 398. The non-livestock-associated MLST types ST1 (2/3), ST97 t2770 (2/2), ST6627 (1/1), and ST7119 (1/1) were more frequently or exclusively found in the failure group. In contrast, isolates of patients with successful decolonization predominantly belonged to community-associated lineages ST6-t304 (4/4), ST8-t008 (5/5), and the livestock-associated clonal cluster 398 (7/7) (Fig. 2).

### Susceptibility and Resistance Genes

**Fig. 3:**
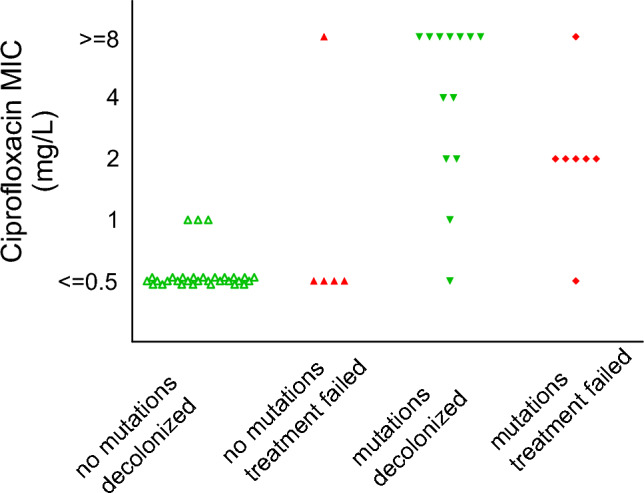
Ciprofloxacin MIC according to MRSA decolonization outcome and mutations associated with ciprofloxacin resistance. MIC range depicted by decolonization outcome and the presence of mutations (in GrlA, GrlB, or GyrA) associated with ciprofloxacin resistance. In the isolates of patients with treatment failure (red, *n* = 12), mutations were identified in 7/12 (58.3%) of the isolates, whereas in the isolates of patients with successful decolonization (green, *n* = 44), mutations were identified in 13/44 (29.5%) isolates. In isolates with high resistance (i.e., MIC >  = 8mg/L) to ciprofloxacin (*n* = 9), multiple mutations were detected (Table S3), except for 1 isolate without mutations

All MRSA isolates tested susceptible for the antibiotics used in the eradication treatments, and this was in line with the sequencing data that showed the absence of acquired resistance genes to these drugs (Table S2). Treatment failure was therefore not the result of resistance against the antibiotics used for the treatment. A significant association was found between ciprofloxacin resistance and failure of eradication (OR 4.20, 95%CI 1.11–15.96, *P* = 0.04) (Table 2). None of the patients had been treated with ciprofloxacin. The ciprofloxacin-resistant isolates belonged to ST5 (5), ST8 (2), ST22 (3), ST30 (1), ST97 (2), ST105 (1), ST398 (1), ST5544 (1), ST7119 (1), and ST8018 (1). In the ciprofloxacin-resistant isolates (*n* = 18), we detected one or more of the associated point-mutations S84L (10/18) in the gyrase GyrA, S80F (14/18) or S80Y (3/18) or E84G (2/18) or I45M (1/18) in the DNA topoisomerase IV GrlA, and P585S (1/18) in GrlB (Table S3). In the isolates of all patients with treatment failure, mutations associated with ciprofloxacin resistance were identified in 7/12 (58%) of the isolates, whereas in the isolates of patients with successful decolonization, these mutations were identified in 13/44 (30%) isolates (Fig. 3). Two isolates with the unique point mutation I45M in GrlA did not show increased MICs to ciprofloxacin. All seven persons with ciprofloxacin-resistant MRSA with failure to eradication treatment were either healthcare workers, or most likely had acquired the MRSA during hospitalization or after medical interventions. Rifampicin resistance-associated point-mutations were found in four isolates (I527L [3/4] and D471Y [1/4] in rpoB). While all four of these isolates had a rifampicin MIC ≤ 0.03, these isolates belonged to four patients with treatment failure (Table S3). No other associations were found between phenotypic antibiotic resistance or resistance genes and failure of eradication treatment (Table 3).

**Table 2:** Phenotypic resistance to antibiotics used in eradication therapy

Antibiotic (R)	Treatment failure	Successful decolonization	*P*
	*N* = 12 (%)	*N* = 44 (%)	
Doxycycline	4 (33.3)	15 (34.1)	1.00
Ciprofloxacin	7 (58.3)	11 (25.0)	0.04
Trimethoprim	0 (0.0)	10 (25.6)	0.09
Cotrimoxazole	0 (0.0)	9 (20.9)	0.18
Clindamycin	6 (50.0)	15 (35.7)	0.50
Rifampicin	0	0	n.a
Mupirocin	0	0	n.a

Phenotypic resistance per antibiotic agent, stratified by decolonization outcome

**Table 3:** Resistance genes

Genes	Treatment failure	Successful decolonization	*P*
	*N* = 12 (%)	*N* = 44 (%)	
erm(C)	4 (33.3)	7 (15.9)	0.22
erm(B)	0 (0)	1 (2.3)	1.00
erm(A)	2 (16.7)	7 (15.9)	1.00
tet(K)	4 (33.3)	13 (29.5)	1.00
tet(L)	1 (8.3)	0 (0)	0.21
tet(M)	0 (0)	5 (11.4)	0.57
tet(S/M)	0 (0)	5 (11.4)	0.57
dfrG	0 (0)	4 (9.1)	0.57
dfrK	0 (0)	1 (2.3)	1.00
fus(B)	0 (0)	1 (2.3)	1.00
fus(C)	2 (16.7)	4 (9.1)	0.60

Resistance genes stratified by decolonization outcome. No genes associated with mupirocin resistance were detected

### Virulence Factors

An overview of the distribution of virulence genes among the patients with eradication failure and patients with successful decolonization is presented in Table 4. No associations were found between virulence genes and failure of eradication. Remarkably, PVL (*lukF_PV* and *lukS_PV*) was found more often in patients with successful decolonization compared to the patients with eradication failure, although non-significant (30% vs 17%, *P* = 0.48). The genes *lukF_PV* and *lukS_PV* and *spIE* were significantly associated with an MRSA infection (*P* < 0.05). The genes *aur, hlgABC, icaACD, setB, setC, hlI, hlII, arcc, aroe, glpf, gmk, pta, tpi, yqil, isaB, lukX, lukY*, and *ebpS* were present in all isolates and were therefore excluded from the analysis. The genes *arc, edinABC, etABD, seb, sec*, and *sed* were only sporadically present and were therefore excluded from the analysis as well.

**Table 4:** Virulence factors

Virulence factors	Genes	Treatment failure	Successful decolonization	*P*
		*N* = 12 (%)	*N* = 44 (%)	
Capsule type 5	*cap5H*	6 (50.0)	28 (63.6)	0.51
	*cap5J*	6 (50.0)	28 (63.6)	0.51
	*cap5K*	6 (50.0)	27 (61.4)	0.52
Capsule type 8	*cap8H*	5 (41.7)	16 (36.4)	0.75
	*cap8I*	6 (50.0)	16 (36.4)	0.51
	*cap8J*	6 (50.0)	16 (36.4)	0.51
	*cap8K*	6 (50.0)	16 (36.4)	0.51
Chemotaxis-inhibiting protein	*chp*	6 (50.0)	27 (61.4)	0.52
Enolase	*eno*	11 (91.7)	44 (100.0)	0.21
Fibrinogen-binding protein	*fib*	11 (91.7)	36 (81.8)	0.67
Leukocidin D/E	*lukD*	8 (66.7)	24 (54.5)	0.53
	*lukE*	8 (66.7)	23 (52.3)	0.52
Panton-Valentine leucocidin	*lukF_PV*	2 (16.7)	13 (29.5)	0.48
	*lukS_PV*	2 (16.7)	13 (29.5)	0.48
Staphylokinase	*sak*	11 (91.7)	34 (77.3)	0.67
Staphylococcal complement inhibitor	*scn*	11 (91.7)	38 (86.4)	1.00
Enterotoxin genes	*seg*	7 (58.3)	20 (45.5)	0.52
	*sei*	7 (58.3)	20 (45.5)	0.52
	*sem*	7 (58.3)	20 (45.5)	0.52
	*sen*	7 (58.3)	18 (40.9)	0.51
	*seo*	7 (58.3)	20 (45.5)	0.52
	*seu*	6 (50.0)	17 (38.6)	0.52
	*seh*	2 (16.7)	1 (2.3)	0.11
	*sek*	2 (16.7)	5 (11.4)	0.64
	*seq*	2 (16.7)	3 (6.8)	0.31
	*sea_sep*	2 (16.7)	9 (20.5)	1.00
	*sej*	1 (8.3)	6 (13.6)	1.00
	*ser*	1 (8.3)	6 (13.6)	1.00
Serine protease A/B/E	*splA*	8 (66.7)	20 (45.5)	0.33
	*splB*	8 (66.7)	21 (47.7)	0.33
	*splE*	5 (41.7)	13 (29.5)	0.50
Toxic shock syndrome toxin-1	*tst1*	0 (0.0)	6 (13.6)	0.32

Virulence factors and genes stratified by decolonization outcome

## Discussion

In this study, we explored associations between MRSA isolate characteristics, genetic determinants, and decolonization outcomes in a Dutch population of MRSA carriers in a tertiary hospital. We found an association of eradication failure with carriage of ciprofloxacin-resistant healthcare-associated lineages, whereas livestock-associated MRSA lineage ST398 and the majority of community-associated MRSA lineages ST6-t304 and ST8-t008 were associated with successful eradication treatment or spontaneous clearance.

The failure rate in eradication treatment of complex MRSA carriers was higher compared to previous reports in Dutch studies [5, 7]. Our study was conducted in the outpatient clinic of a tertiary hospital, with consequently a more than average representation of healthcare workers or patients with an extensive history of hospitalizations. Such patients mainly carry healthcare-associated MRSAs, that are adapted to survive under harsh nosocomial conditions and antibiotic exposure.

In our study, we found an association between ciprofloxacin resistance and failure in eradication treatment. Remarkably, none of the patients had been treated with ciprofloxacin. The ciprofloxacin-resistant MRSAs in our study belonged to various lineages, including five isolates of the healthcare-associated ST5 lineage with single amino acid substitution in GrlA S80F. The mutation in this healthcare-associated lineage, and its association with fluoroquinolone resistance and the presence of virulence genes as enterotoxins, β-hemolysin converting phage, and leucocidins has been described previously [21]. The resistance to fluoroquinolones is generally high in healthcare-associated MRSA [22]. Successful hospital-adapted ciprofloxacin-resistant lineages have emerged among several nosocomial species as *E. coli*, *K. pneumoniae*, vancomycin-resistant *E. faecium*, and MRSA. These lineages have acquired stable point-mutations in gyrase and/or topoisomerase IV enzymes [23]. It is unsure what drives this evolution, besides the exposure to fluoroquinolones.

Both tolerance and persistence have been reported in low-level ciprofloxacin-resistant *E. coli*, allowing to survive exposure to therapeutic concentrations of ciprofloxacin [24]. In tolerance, bacterial cells survive using a “hibernation mode,” in which the cell cycle and metabolism are temporarily stopped, preventing killing by antibiotics. In persistence, a bacterial subpopulation is able to survive antibiotic exposure [25]. Cross-tolerance to multi-drugs has been reported, but does not necessarily occur in all tolerant isolates and is dependent on antibiotic regimen and duration of exposure [26]. To the best of our knowledge, no studies have reported cross-tolerance in low-level ciprofloxacin-resistant *S. aureus* isolates to the antibiotic regimens in MRSA eradication used in this study. Therefore, the explanation for the association found in our study remains uncertain. Potentially, healthcare-associated MRSAs are more prone to failure of eradication treatment, and ciprofloxacin resistance may be a biomarker for these difficult-to-treat lineages.

The recent finding of association between *chp* and carriage duration was not found in our study [8]. Compared to the Danish study, our patient population had more healthcare-associated MRSA. Also, there is large heterogeneity in the Danish and Dutch MRSA treatment guidelines. The main difference is the more general use of two systemic antibiotics in the Netherlands, compared to sporadic systemic treatment in Denmark.

Two studies, in Denmark and Sweden, reported that PVL-positive isolates had a higher eradication success rate [15, 27]. We also found a higher (non-significant) rate of PVL-positive isolates in the successful eradication group, mainly belonging to the CA-MRSA linages ST30 and ST8-t008. However, associations do not necessarily reflect an etiologic cause, but can also reflect markers or confounders. We postulate that PVL is a marker of certain non-healthcare-associated MRSA lineages that are easier to eradicate, rather than a direct positive effect of the PVL toxin to eradication outcomes.

There are multiple factors of potential influence on MRSA eradication outcome. Carriers can reacquire MRSA isolates from contamination in their environment, or by positive household members. The eradication treatment of patients in this study was performed in a specialized outpatient clinic setting, following the Dutch eradication protocol [16]. Several measures are taken to prevent reacquisition, such as simultaneous treatment of positive household members and hygienic instructions. Isolate characteristics may also play a role in the risk of spread and reacquisition of MRSA. Hetem et al. showed that in a hospital setting, the transmission of livestock-associated MRSA was 4.4 times lower compared to non-livestock-associated MRSA isolates [12]. In general, MRSA isolates can be able to survive antibiotic exposure, despite having a MIC indicating susceptibility to the antibiotic agent. Our study showed that the antibiotic treatment failure is not explained by the common acquired resistance genes related to resistance, of which the presence or absence corresponded to the phenotypic susceptibility in all isolates. However, alternative survival mechanisms to antibiotic exposure, such as tolerance and persistence, are not detectable by measuring MICs. Other potential factors influencing MRSA eradication outcome, e.g., therapy incompliance and host genetics [28], were not assessed in our study.

There are some limitations of this study. It is a single-center study with a small sample size, a heterogeneous population, and a limited number of failed treatments. In addition, we did not always confirm that treatment failure was caused by the same clone, or acquisition of a different MRSA. However, given the very low prevalence of MRSA in the Netherlands, this would be highly unlikely. Furthermore, we did not correct for multiple testing. However, since it is an explorative study in a relatively undiscovered subject, we believe the results are still valid and useful in targeting future research. For this explorative purpose, we focused on pathogen factors and only added a limited number of host characteristics (i.e., sex, age, and complicated versus uncomplicated carriership). Other host factors—including host genetics—may influence the risk of treatment failure as well. Lastly, we investigated genes with a previously reported role in virulence. Future genome-wide association studies could perhaps identify signatures with novel genetic factors implicated in intracellular survival and biofilm formation that predict eradication failure. However, this requires a larger and preferably prospective data set.

In conclusion, this explorative study showed a higher eradication failure rate in complicated MRSA carriers with ciprofloxacin-resistant MRSA lineages, which are predominantly healthcare-associated. In contrast, carriers of livestock-associated MRSA and the major community-associated ST8 and ST6 lineages were generally successfully decolonized. Further studies are warranted to confirm the higher eradication failure risk of ciprofloxacin-resistant lineages, and identify the underlying mechanisms. The identification of lineages that are prone to eradication failure is of clinical relevance, since it could influence the initiation and monitoring of MRSA eradication therapy.

### Supplementary Information

Below is the link to the electronic supplementary material.

Supplementary file1 (DOCX 72 KB)

## Data Availability

The MRSA sequence data have been deposited in ENA under project number PRJEB59407.
