# Supplementary material for: Genetic Determinants in MRSA Carriage and Their Association with Decolonization Outcome
Source: Curr Microbiol. 2024 Jan 13;81(2):63. doi: 10.1007/s00284-023-03581-w (PMC10787693; doi:10.1007/s00284-023-03581-w)
Supplement: Supplementary file 1 — Supplementary file1 (DOCX 72 KB) [file 284_2023_3581_MOESM1_ESM.docx]

Supplementary data

**Table S1.**  Isolate characteristics

| Isolate | MLST | Spa | MIC VA | MIC DX | MIC CI | Disc TR | MIC  TR | MIC SXT | MIC RA | MIC CL | Res VA | Res DX | Res CI | Res TR | Res SXT | Res RA | Res CL |
| --- | --- | --- | --- | --- | --- | --- | --- | --- | --- | --- | --- | --- | --- | --- | --- | --- | --- |
| 37 | 97 | 2770 | ≤ 0.5 | ≥ 16.0 | 2 | 24 |  | ≤ 10.0 | ≤ 0.03 | 0.25 | S | R | R | S | S | S | S |
| 507 | 22 | 2933 | ≤ 0.5 | ≤ 1.0 | ≤ 0.5 | - | 6 | 160 | ≤ 0.03 | 0.25 | S | S | S | R* | R | S | S |
| 690 | 398 | 011 | ≤ 0.5 | ≥ 16.0 | ≤ 0.5 | 6 |  | 80 | ≤ 0.03 | ≥ 4.0 | S | R | S | R | R | S | R |
| 720 | 5 | 002 | ≤ 0.5 | ≤ 1.0 | 2 | - |  | ≤ 10.0 | ≤ 0.03 | 0.25 | S | S | R |  | S | S | R |
| 1475 | 5 | 062 | 1 | ≤ 1.0 | ≤ 0.5 | 6 |  | ≤ 10.0 | ≤ 0.03 | 0.25 | S | S | S | R | S | S | S |
| 2213 | 30 | 019 | 1 | ≥ 16.0 | ≤ 0.5 | 21 |  | ≤ 10.0 | ≤ 0.03 | 0.25 | S | R | S | S | S | S | S |
| 2223 | 88 | 3622 | 1 | ≤ 1.0 | ≤ 0.5 | 25 |  | ≤ 10.0 | ≤ 0.03 | 0.25 | S | S | S | S | S | S | R |
| 2285 | 7 | 091 | 1 | ≤ 1.0 | ≤ 0.5 | 20 |  | ≤ 10.0 | ≤ 0.03 | 0.25 | S | R | S | S | S | S | R |
| 2315 | 398 | 011 | ≤ 0.5 | ≥ 16.0 | 1 | 25 |  | ≤ 10.0 | ≤ 0.03 | ≥ 4.0 | S | R | S | S | S | S | R |
| 2392 | 8 | 008 | ≤ 0.5 | ≤ 1.0 | ≤ 0.5 | 21 |  | ≤ 10.0 | ≤ 0.03 | 0.25 | S | S | S | S | S | S | S |
| 2478 | 30 | 2217 | ≤ 0.5 | ≤ 1.0 | 1 | 17 |  | ≤ 10.0 | ≤ 0.03 | 0.25 | S | S | S | S | S | S | S |
| 2654 | 5 | 002 | 1 | ≤ 1.0 | 2 | 23 |  | ≤ 10.0 | ≤ 0.03 | 0.25 | S | S | R | S | S | S | R |
| 2673 | 398 | 108 | ≤ 0.5 | ≥ 16.0 | ≤ 0.5 | - |  | ≤ 10.0 | ≤ 0.03 | 0.25 | S | R | S |  | S | S | S |
| 2703 | 398 | 011 | 1 | ≥ 16.0 | ≥ 8.0 | 23 |  | ≤ 10.0 | ≤ 0.03 | 0.25 | S | R | R | S | S | S | S |
| 2855 | 1535 | 084 | 1 | ≥ 16.0 | ≤ 0.5 | 23 |  | ≤ 10.0 | ≤ 0.03 | 0.5 | S | R | S | S | S | S |  |
| 2866 | 5 | 002 | ≤ 0.5 | ≤ 1.0 | 1 | 22 |  | ≤ 10.0 | ≤ 0.03 | 0.25 | S | S | S | S | S | S | R |
| 2879 | 5 | 6212 | 1 | ≤ 1.0 | 2 | 23 |  | ≤ 10.0 | ≤ 0.03 | 0.25 | S | S | R | S | S | S | R |
| 2905 | 5 | 311 | ≤ 0.5 | ≤ 1.0 | 4 | 6 |  | 160 | ≤ 0.03 | 0.25 | S | S | R | R | R | S | S |
| 2970 | 80 | 044 | ≤ 0.5 | ≥ 16.0 | ≤ 0.5 | 23 |  | ≤ 10.0 | ≤ 0.03 | 0.25 | S | R | S | S | S | S | S |
| 2980 | 398 | 011 | ≤ 0.5 | ≥ 16.0 | ≤ 0.5 | 6 |  | ≥ 320.0 | ≤ 0.03 | 0.25 | S | R | S | R | R | S | S |
| 3025 | 5 | 002 | 1 | ≤ 1.0 | 2 | 22 |  | ≤ 10.0 | ≤ 0.03 | 0.25 | S | S | R | S | S | S | R |
| 3260 | 6 | 304 | 1 | ≤ 1.0 | ≤ 0.5 | 22 |  | ≤ 10.0 | ≤ 0.03 | 0.25 | S | S | S | S | S | S | R |
| 3460 | 8017 | 442 | 1 | ≤ 1.0 | ≤ 0.5 | 21 |  | ≤ 10.0 | ≤ 0.03 | 0.25 | S | S | S | S | S | S | S |
| 3464 | 22 | 223 | ≤ 0.5 | ≥ 16.0 | ≤ 0.5 | - |  | 160 | ≤ 0.03 | 0.25 |  | R | S |  |  |  | S |
| 3488 | 8 | 008 | ≤ 0.5 | ≤ 1.0 | ≤ 0.5 | 19 |  | ≤ 10.0 | ≤ 0.03 | 0.25 | S | S | S | S | S | S | S |
| 3839 | 8 | 008 | 1 | ≥ 16.0 | ≥ 8.0 | 19 |  | ≤ 10.0 | ≤ 0.03 | ≥ 4.0 | S | R | R | S | S | S | R |
| 4211 | 22 | 294 | 1 | ≤ 1.0 | ≥ 8.0 | 20 |  | ≤ 10.0 | ≤ 0.03 | 0.25 | S | S | R | S | S | S | S |
| 4998 | 4811 | 330 | ≤ 0.5 | ≤ 1.0 | ≤ 0.5 | 19 |  | ≤ 10.0 | ≤ 0.03 | 0.25 | S | S | S | S | S | S | R |
| 5308 | 6 | 304 | 1 | ≤ 1.0 | ≤ 0.5 | 21 |  | ≤ 10.0 | ≤ 0.03 | 0.25 | S | S | S | S | S | S | S |
| 5606 | 22 | 022 | ≤ 0.5 | ≤ 1.0 | ≥ 8.0 | 27 |  | ≤ 10.0 | ≤ 0.03 | ≥ 4.0 | S | S | R | S | S | S | R |
| 5940 | 22 | 309 | ≤ 0.5 | ≤ 1.0 | ≥ 8.0 | 6 |  | 80 | ≤ 0.03 | 0.25 | S | S | R | R | R | S | S |
| 6118 | 1 | 127 | 1 | ≥ 16.0 | ≤ 0.5 | 23 |  | ≤ 10.0 | ≤ 0.03 | 0.25 | S | S | S | S | S | S | R |
| 6330 | 105 | 002 | 1 | ≤ 1.0 | ≥ 8.0 | 24 |  | ≤ 10.0 | ≤ 0.03 | ≥ 4.0 | S | S | R | S | S | S | R |
| 6441 | 22 | 223 | ≤ 0.5 | ≤ 1.0 | ≤ 0.5 | 6 |  | ≥ 320.0 | ≤ 0.03 | 0.25 | S | S | S | R | R | S | S |
| 6779 | 1153 | 903 | ≤ 0.5 | ≤ 1.0 | ≤ 0.5 | 26 |  | ≤ 10.0 | ≤ 0.03 | 0.25 | S | S | S | S | S | S | S |
| 6826 | 6 | 304 | ≤ 0.5 | ≤ 1.0 | ≤ 0.5 | 23 |  | ≤ 10.0 | ≤ 0.03 | 0.25 | S | S | S | S | S | S | S |
| 7211 | 4131 | 034 | ≤ 0.5 | ≥ 16.0 | ≤ 0.5 | - |  | ≤ 10.0 | ≤ 0.03 | ≥ 4.0 | S | R | S |  | S | S | R |
| 7261 | 1232 | 034 | ≤ 0.5 | ≥ 16.0 | ≤ 0.5 | 25 |  | ≤ 10.0 | ≤ 0.03 | ≥ 4.0 | S | R | S | S | S | S | R |
| 7346 | 8015 | 026 | ≤ 0.5 | ≤ 1.0 | ≤ 0.5 | 21 |  | ≤ 10.0 | ≤ 0.03 | 0.25 | S | S | S | S | S | S | R |
| 7435 | 8018 | 442 |  | ≤ 1.0 | ≥ 8.0 | 22 |  | ≤ 10.0 | ≤ 0.03 | 0.25 |  | S | R | S | S | S | S |
| 7450 | 6 | 304 | ≤ 0.5 | ≤ 1.0 | ≤ 0.5 | 22 |  | ≤ 10.0 | ≤ 0.03 | 0.25 | S | S | S | S | S | S | R |
| 7473 | 97 | 2770 | 1 | ≥ 16.0 | 2 | 19 |  | ≤ 10.0 | ≤ 0.03 | 0.25 | S | R | R | S | S | S | S |
| 7761 | 30 | 019 | 1 | ≥ 16.0 | ≤ 0.5 | 20 |  | ≤ 10.0 | ≤ 0.03 | 0.25 | S | R | S | S | S | S | S |
| 8148 | 1535 | 084 | 1 | ≤ 1.0 | 1 | 21 |  | ≤ 10.0 | ≤ 0.03 | 0.25 | S | S | S | S | S | S | S |
| 8178 | 5544 | 1081 | 1 | ≥ 16.0 | ≥ 8.0 | 21 |  | ≤ 10.0 | ≤ 0.03 | 0.5 | S | R | R | S | S | S |  |
| 8244 | 8 | 008 | 1 | ≤ 1.0 | ≤ 0.5 | 22 |  | ≤ 10.0 | ≤ 0.03 | 0.25 | S | S | S | S | S | S | S |
| 8349 | 8 | 008 | ≤ 0.5 | ≤ 1.0 | ≥ 8.0 | 21 |  | ≤ 10.0 | ≤ 0.03 | 0.5 | S | S | R | S | S | S | S |
| 8413 | 30 | 363 | 1 | ≤ 1.0 | 4 | 6 |  | ≥ 320.0 | ≤ 0.03 | 0.25 | S | S | R | R | R | S | S |
| 8578 | 5 | 1062 | 1 | ≤ 1.0 | ≤ 0.5 | 6 |  | ≥ 320.0 | ≤ 0.03 | 0.25 | S | S | S | R | R | S | S |
| 8852 | 6627 | 304 | 1 | ≤ 1.0 | ≤ 0.5 | 22 |  | ≤ 10.0 | ≤ 0.03 | 0.25 | S | S | S | S | S | S | S |
| 8935 | 1 | 127 | 1 | ≥ 16.0 | ≤ 0.5 | 21 |  | ≤ 10.0 | ≤ 0.03 | 0.25 | S | R | S | S | S | S | R |
| 9038 | 8016 | 304 | 1 | ≤ 1.0 | ≤ 0.5 | 23 |  | ≤ 10.0 | ≤ 0.03 | 0.25 | S | S | S | S | S | S | S |
| 9601 | 7119 | 132 | ≤ 0.5 | ≤ 1.0 | 2 | 23 |  | ≤ 10.0 | ≤ 0.03 | 0.25 | S | S | R | S | S | S | S |
| 9941 | 22 | 790 | 1 | ≤ 1.0 | ≤ 0.5 | - |  | ≤ 10.0 | ≤ 0.03 | 0.25 | S | S | S |  | S | S | S |
| 302760 | 22 | 2251 | 1 | ≤ 1.0 | ≤ 0.5 | 6 |  | ≥ 320.0 | ≤ 0.03 | 0.25 | S | S | S | R | R | S | S |
| 502760 | 1 | 127 | 1 | ≥ 16.0 | ≤ 0.5 | 17 |  | ≤ 10.0 | ≤ 0.03 | 0.25 | S | R | S | S | S | S | R |

*Legend:* MIC is in mg/L. VA: vancomycin, DX: doxycycline, CI: ciprofloxacin, TR: trimethoprim, SXT: trimethoprim/sulfamethoxazole, SF: sulphonamides, RA: rifampicin, CL: clindamycin, MP: mupirocin. Res = resistance. S/R: susceptible/intermediate/resistant. *etest MIC: 6mg/L.

**Table S2.** Characteristics of isolates of patients that were treated with systemic antibiotics

| **Isolate** | **Treatment regimen/ duration** | **MIC/ susceptibility**  **resistance genes DX** | **MIC/ susceptibility**  **resistance genes VA** | **Disk zone/ susceptibility**  **resistance genes TR** | **MIC/ susceptibility SXT**  **resistance genes SF** | **MIC/ susceptibility**  **resistance genes RA** | **MIC/ susceptibility**  **resistance genes CL** | **Outcome*** |
| --- | --- | --- | --- | --- | --- | --- | --- | --- |
| 7346 | DX RA MP / 7 | ≤ 1.0 / S | ≤ 0.5 / S | 21 / S | ≤ 10.0 /S | ≤ 0.03/ S | 0.25/ R | Failure |
|  |  | - | - | - | - | - | erm(C) |  |
| 0037 | TR RA MP / 7 | ≥ 16.0 / R | ≤ 0.5 / S | 24/ S | ≤ 10.0 / S | ≤ 0.03/ S | 0.25 / S | Failure |
|  |  | tet(K) | - | - | - | - | - |  |
| 2654 | DX RA MP / 7 | ≤ 1.0 / S | 1 / S | 23 / S | ≤ 10.0 / S | ≤ 0.03 / S | 0.25 / R | Failure |
|  |  | - | - | - | - | - | erm(A) |  |
| 502760 | SXT RA MP / 14 | ≥ 16.0 / R | 1 / S | 17 / S | ≤ 10.0 / S | ≤ 0.03 / S | 0.25 / R | Failure |
|  |  | tet(L) | - | - | - | - | erm(C) |  |
| 3025 | DX RA MP / 7 | ≤ 1.0 / S | ≤ 0.5 / S | 22 / S | ≤ 10.0 / S | ≤ 0.03 / S | 0.25 / R | Failure |
|  |  | - | - | - | - | - | erm(A) |  |
| 5606 | SXT RA MP / 7 | ≤ 1.0 / S | 1 / S | 27 / S | ≤ 10.0 / S | ≤ 0.03 / S | ≥ 4.0 / R | Failure |
|  |  | - | - | - | - | - | erm(C) |  |
| 6118 | TR RA MP / 7 | ≥ 16.0 / R | 1 / S | 23 / S | ≤ 10.0 / S | ≤ 0.03 / S | 0.25 / R | Failure |
|  |  | tet(K) | - | - | - | - | erm(C) |  |
| 7435 | TR RA MP / 7 | ≤ 1.0 / S | - / - | - / S | ≤ 10.0 / S | ≤ 0.03 / S | 0.25 / S | Failure |
|  |  | - | - | - | - | - | - |  |
| 7473 | TR RA MP / 7 | ≥ 16.0 / R | 1 / S | 19 / S | ≤ 10.0 / S | ≤ 0.03 / S | 0.25 / S | Failure |
|  |  | tet(K) | - | - | - | - | - |  |
| 7761 | SXT RA MP / 7 | ≥ 16.0 / R | 1 / S | 20 / S | ≤ 10.0 / S | ≤ 0.03 / S | 0.25 / S | Failure |
|  |  | tet(K) | - | - | - | - | - |  |
| 8852 | SXT RA MP / 7 | ≤ 1.0 / S | 1 / S | 22 / S | ≤ 10.0 / S | ≤ 0.03 / S | 0.25 / S | Failure |
|  |  | - | - | - | - | - | - |  |
| 0507 | DX RA MP / 7 | ≤ 1.0 / S | ≤ 0.5 / S | - / R | 160 / R | ≤ 0.03 / S | 0.25 / S | Success |
|  |  | - | - | - | - | - | - |  |
| 1475 | DX RA MP / 14 | ≤ 1.0 / S | 1 / S | 6 / R | ≤ 10.0 / S | ≤ 0.03 / S | 0.25 / S | Success |
|  |  | - | - | - | - | - | - |  |
| 2285 | DX RA MP / 14 | ≤ 1.0 / R | 1 / S | 20 / S | ≤ 10.0 / S | ≤ 0.03 / S | 0.25 / R | Success |
|  |  | - | - | - | - | - | - |  |
| 3460 | SXT RA MP / 7 | ≤ 1.0 / R | 1 / S | 21 / - | ≤ 10.0 / S | ≤ 0.03 / S | 0.25 / S | Success |
|  |  | - | - | - | - | - | - |  |
| 5940 | DX RA MP / 7 | ≤ 1.0 / S | ≤ 0.5 / S | - / R | 80 / R | ≤ 0.03 / S | 0.25 / S | Success |
|  |  | - | - | - | - | - | - |  |
| 8413 | DX RA MP / 7 | ≤ 1.0 / S | 1 / S | 6 / R | ≥ 320.0 / R | ≤ 0.03 / S | 0.25 / S | Success |
|  |  | - | - | dfrG | - | - | - |  |
| 2213 | SXT RA MP / 7 | ≥ 16.0 / R | 1 / S | 21 / S | ≤ 10.0 / S | ≤ 0.03 / S | 0.25 / S | Success |
|  |  | tet(K) | - | - | - | - | - |  |
| 2223 | SXT RA MP / 7 | ≤ 1.0 / S | 1 / S | 25 / S | ≤ 10.0 / S | ≤ 0.03 / S | 0.25 / R | Success |
|  |  | - | - | - | - | - | erm(C) |  |
| 2392 | CL RA MP / 14 | ≤ 1.0 / S | ≤ 0.5 / S | 21 / S | ≤ 10.0 / S | ≤ 0.03 / S | 0.25 / S | Success |
|  |  | - | - | - | - | - | - |  |
| 302760 | CL RA MP / 7 | ≤ 1.0 / S | 1 / S | 6 / R | ≥ 320.0 / R | ≤ 0.03 / S | 0.25 / S | Success |
|  |  | - | - | - | - | - | - |  |
| 2879 | TR RA MP / 7 | ≤ 1.0 / S | 1 / S | 23 / S | ≤ 10.0 / S | ≤ 0.03 / S | 0.25 / R | Success |
|  |  | - | - | - | - | - | erm(A) |  |
| 3260 | DX RA MP / 7 | ≤ 1.0 / S | 1 / S | 22 / S | ≤ 10.0 / S | ≤ 0.03 / S | 0.25 / R | Success |
|  |  | - | - | - | - | - | erm(C) |  |
| 3488 | CL RA MP / 7 | ≤ 1.0 / S | ≤ 0.5 / S | 19 / S | ≤ 10.0 / S | ≤ 0.03 / S | 0.25 / S | Success |
|  |  | - | - | - | - | - | - |  |
| 3839 | TR RA MP / 7 | ≥ 16.0 / R | 1 / S | 19 / S | ≤ 10.0 / S | ≤ 0.03 / S | ≥ 4.0 / R | Success |
|  |  | tet(K) | - | - | - | - | erm(C) |  |
| 4998 | DX RA MP / 7 | ≤ 1.0 / S | ≤ 0.5 / S | 19 / S | ≤ 10.0 / S | ≤ 0.03 / S | 0.25 / R | Success |
|  |  | - | - | - | - | - | erm(A) |  |
| 5308 | DX RA MP / 7 | ≤ 1.0 / S | 1 / S | 21 / S | ≤ 10.0 / S | ≤ 0.03 / S | 0.25 / S | Success |
|  |  | - | - | - | - | - | - |  |
| 6826 | DX RA MP / 7 | ≤ 1.0 / S | ≤ 0.5 / S | 23 / S | ≤ 10.0 / S | ≤ 0.03 / S | 0.25 / S | Success |
|  |  | - | - | - | - | - | - |  |
| 7211 | SXT RA MP / 14 | ≥ 16.0 / R | ≤ 0.5 / S | - / S | ≤ 10.0 / S | ≤ 0.03 / S | ≥ 4.0 / R | Success |
|  |  | tet(K) | - | - | - | - | erm(A) |  |
| 7261 | SXT RA MP / 7 | ≥ 16.0 / R | ≤ 0.5 / S | 25 / S | ≤ 10.0 / S | ≤ 0.03 / S | ≥ 4.0 / R | Success |
|  |  | tet(K) | - | - | - | - | erm(A) |  |
| 7450 | TR RA MP / 7 | ≤ 1.0 / S | ≤ 0.5 / S | 22 / S | ≤ 10.0 / S | ≤ 0.03 / S | 0.25 / R | Success |
|  |  | - | - | - | - | - | erm(C) |  |
| 8178 | SXT RA MP / 7 | ≥ 16.0 / R | 1 / S | 21 / S | ≤ 10.0 / S | ≤ 0.03 / S | 0.5 / I | Success |
|  |  | tet(K) | - | - | - | - | - |  |
| 8244 | VA CL MP / 7 | ≤ 1.0 / S | 1 / S | 22 / S | ≤ 10.0 / S | ≤ 0.03 / S | 0.25 / S | Success |
|  |  | - | - | - | - | - | - |  |
| 8935 | SXT RA MP / 7 | ≥ 16.0 / R | 1 / S | 21 / S | ≤ 10.0 / S | ≤ 0.03 / S | 0.25 / R | Success |
|  |  | tet(K) | - | - | - | - | erm(C) |  |

*Legend:* Treatment duration is in days. MIC is in mg/L. Disc zone is in millimetres. VA: vancomycin, DX: doxycycline, TR: trimethoprim, SXT: trimethoprim/sulfamethoxazole, SF: sulphonamides, RA: rifampicin, CL: clindamycin, MP: mupirocin. S/I/R: susceptible/intermediate/resistant. *Success = successful decolonization. Failure = failure of eradication treatment.

**Table S3.** Point mutations associated with ciprofloxacin and rifampicin resistance and MICs.

| **Isolate** | **Outcome** | **MIC ciprofloxacin** | **Ciprofloxacin-resistance associated mutations** | **MIC rifampicin** | **Rifampicin-resistance associated mutations** |
| --- | --- | --- | --- | --- | --- |
| 37 | Failure | 2 | S80F grlA | ≤ 0.03 | D471Y rpoB |
| 507 | Success | ≤ 0.5 |  | ≤ 0.03 |  |
| 690 | Success | ≤ 0.5 |  | ≤ 0.03 |  |
| 720 | Success | 2 | S80F grlA | ≤ 0.03 |  |
| 1475 | Success | ≤ 0.5 |  | ≤ 0.03 |  |
| 2213 | Success | ≤ 0.5 |  | ≤ 0.03 |  |
| 2223 | Success | ≤ 0.5 |  | ≤ 0.03 |  |
| 2285 | Success | ≤ 0.5 |  | ≤ 0.03 |  |
| 2315 | Success | 1 |  | ≤ 0.03 |  |
| 2392 | Success | ≤ 0.5 |  | ≤ 0.03 |  |
| 2478 | Success | 1 |  | ≤ 0.03 |  |
| 2654 | Failure | 2 | S80F grlA | ≤ 0.03 |  |
| 2673 | Success | ≤ 0.5 |  | ≤ 0.03 |  |
| 2703 | Success | ≥ 8.0 | S80F grlA  S84L gyrA | ≤ 0.03 |  |
| 2855 | Success | ≤ 0.5 |  | ≤ 0.03 |  |
| 2866 | Success | 1 | S80F grlA | ≤ 0.03 |  |
| 2879 | Success | 2 | S80F grlA | ≤ 0.03 |  |
| 2905 | Success | 4 | S80F grlA  S84L gyrA | ≤ 0.03 |  |
| 2970 | Success | ≤ 0.5 |  | ≤ 0.03 |  |
| 2980 | Success | ≤ 0.5 |  | ≤ 0.03 |  |
| 3025 | Failure | 2 | S80F grlA | ≤ 0.03 |  |
| 3260 | Success | ≤ 0.5 |  | ≤ 0.03 |  |
| 3460 | Success | ≤ 0.5 |  | ≤ 0.03 |  |
| 3464 | Success | ≤ 0.5 |  | ≤ 0.03 |  |
| 3488 | Success | ≤ 0.5 |  | ≤ 0.03 |  |
| 3839 | Success | ≥ 8.0 | S84L gyrA  S80Y grlA | ≤ 0.03 |  |
| 4211 | Success | ≥ 8.0 | S84L gyrA  S80Y grlA | ≤ 0.03 |  |
| 4998 | Success | ≤ 0.5 | I45M grlA | ≤ 0.03 |  |
| 5308 | Success | ≤ 0.5 |  | ≤ 0.03 |  |
| 5606 | Failure | ≥ 8.0 | S80F grlA  S84L gyrA  P585S grlB | ≤ 0.03 |  |
| 5940 | Success | ≥ 8.0 | S80F grlA  S84L gyrA | ≤ 0.03 |  |
| 6118 | Failure | ≤ 0.5 |  | ≤ 0.03 |  |
| 6330 | Success | ≥ 8.0 | S80y grlA  S84L gyrA  E84G grlA | ≤ 0.03 |  |
| 6441 | Success | ≤ 0.5 |  | ≤ 0.03 |  |
| 6779 | Success | ≤ 0.5 |  | ≤ 0.03 |  |
| 6826 | Success | ≤ 0.5 |  | ≤ 0.03 |  |
| 7211 | Success | ≤ 0.5 |  | ≤ 0.03 |  |
| 7261 | Success | ≤ 0.5 |  | ≤ 0.03 |  |
| 7346 | Failure | ≤ 0.5 | I45M grlA | ≤ 0.03 |  |
| 7435 | Failure | ≥ 8.0 |  | ≤ 0.03 |  |
| 7450 | Success | ≤ 0.5 |  | ≤ 0.03 |  |
| 7473 | Failure | 2 | S80F grlA | ≤ 0.03 | I527L rpoB |
| 7761 | Failure | ≤ 0.5 |  | ≤ 0.03 |  |
| 8148 | Success | 1 |  | ≤ 0.03 |  |
| 8178 | Success | ≥ 8.0 | S80F grlA  S84L gyrA  E84G grlA | ≤ 0.03 |  |
| 8244 | Success | ≤ 0.5 |  | ≤ 0.03 |  |
| 8349 | Success | ≥ 8.0 | S80F grlA  S84L gyrA | ≤ 0.03 |  |
| 8413 | Success | 4 | S80F grlA  S84L gyrA | ≤ 0.03 |  |
| 8578 | Success | ≤ 0.5 |  | ≤ 0.03 |  |
| 8852 | Failure | ≤ 0.5 |  | ≤ 0.03 | I527L rpoB |
| 8935 | Success | ≤ 0.5 |  | ≤ 0.03 |  |
| 9038 | Success | ≤ 0.5 |  | ≤ 0.03 |  |
| 9601 | Failure | 2 | S80F grlA  I45M grlA | ≤ 0.03 | I527L rpoB |
| 9941 | Success | ≤ 0.5 |  | ≤ 0.03 |  |
| 302760 | Success | ≤ 0.5 |  | ≤ 0.03 |  |
| 502760 | Failure | ≤ 0.5 |  | ≤ 0.03 |  |

*Legend:* MIC is in mg/L.
